# Three-dimensional rotating flow of carbon nanotubes with Darcy-Forchheimer porous medium

**DOI:** 10.1371/journal.pone.0179576

**Published:** 2017-07-07

**Authors:** Tasawar Hayat, Farwa Haider, Taseer Muhammad, Ahmed Alsaedi

**Affiliations:** 1 Department of Mathematics, Quaid-I-Azam University, Islamabad, Pakistan; 2 Nonlinear Analysis and Applied Mathematics (NAAM) Research Group, Department of Mathematics, Faculty of Science, King Abdulaziz University, Jeddah, Saudi Arabia; North China Electric Power University, CHINA

## Abstract

Here we are concerned with the Darcy-Forchheimer three-dimensional flow of carbon nanotubes in a rotating frame. Flow is generated by stretching of the surface. Xue model is adopted for nanofluid transport mechanism. Results for single wall carbon nanotubes (SWCNTs) and multi wall carbon nanotubes are achieved and compared. Flow saturating porous space obeys Darcy-Forchheimer expression. Boundary layer approximations are invoked to simplify governing partial differential system. Optimal homotopy analysis method (OHAM) is utilized for solutions of governing model. The optimal values of auxiliary parameters are computed. Plots have been displayed in order to analyze how the velocities and temperature fields get affected by various flow parameters. Skin-friction coefficients and local Nusselt number are presented through numerical data for both SWCNTs and MWCNTs. Moreover the skin-friction coefficients and local Nusselt number are enhanced for larger values of nanoparticles volume fraction.

## 1. Introduction

Carbon nanotubes are hexagonally shaped arrangements of carbon atoms that have been rolled into tubes. Carbon nanotubes were discovered in 1991 by Iijima. Carbon nanotubes have high thermal conductivity, exceptional mechanical strength and exceptional corrosion resistance. Their novel properties make them very useful in applications like microwave amplifier, nanotube sensors, nanotube transistors, hand held X-ray, field emission display, solar cell, lithium ion batteries and chemical sensors. Carbon nanotubes are mainly classified into two types namely, single walled carbon nanotubes (SWCNTs) and multi walled carbon nanotubes (MWCNTs). Choi et al. [1] analyzed anomalous thermal conductivity enhancement in the nanotube suspensions. They found that the nanotubes provide the highest thermal conductivity enhancement. Ramasubramaniam et al. [2] addressed homogeneous carbon nanotubes/polymer composites for electrical applications. Xue [3] provided a model for carbon nanotube-based composites. Heat transfer analysis of aqueous suspensions for carbon nanotubes is studied by Ding et al. [4]. Kamali and Binesh [5] performed numerical investigation of heat transfer enhancement using carbon nanotube. The authors have considered non-Newtonian materials as a base fluid. Wang et al. [6] investigated heat transfer and pressure drop aspects in nanofluids flows containing carbon nanotubes. Safaei et al. [7] examined heat transfer enhancement in a forward-facing contracting channel using FMWCNT nanofluids. Hayat et al. [8] explored Newtonian heating in stagnation point flow of carbon nanotubes with homogeneous and heterogeneous reactions. Ellahi et al. [9] discussed natural convective boundary layer flow of nanofluid with single and multi-walled carbon nanotubes. Karimipour et al. [10] imposed the uniform heat flux to develop MHD forced convective flow of water/FMWNT carbon nanotubes in a microchannel. Hayat et al. [11] discussed the homogeneous and heterogeneous reactions in the flow of nanofluids by a nonlinear stretchable surface with variable thickness. Imtiaz et al. [12] examined convective flow of carbon nanotubes between rotating stretchable disks with thermal radiation. Haq et al. [13] addressed MHD pulsatile flow of engine oil based carbon nanotubes between two concentric cylinders. Hayat et al. [14] discussed simultaneous effects of homogeneous-heterogeneous reactions and convective conditions in boundary-layer flow of carbon nanotubes. Khan et al. [15] provided a numerical study for three-dimensional squeezing flow of carbon nanotubes in a rotating channel. Iqbal et al. [16] examined stagnation-point flow of carbon nanotubes in the presence of induced magnetic field. Recently Hayat et al. [17] studied Darcy-Forchheimer flow of carbon nanotubes due to a rotating disk.

Recently the researchers are engaged in analyzing the rotating flows near stretchable or inextensible boundaries due to its vast applications in rotor-stator system, food processing, rotating machinery, disk cleaners, gas turbine design and many others. Wang [18] provided perturbation solutions for rotating fluid flow past a stretchable surface. Takhar et al. [19] examined the magnetic field characteristics in rotating flow bounded by a stretchable surface. Nazar et al. [20] discussed time-dependent rotating flow past an impulsively deforming surface. Keller-box technique is implemented for the solution. Javed et al. [21] provided locally similar solutions for rotating fluid flow past an exponentially deforming surface. Zaimi et al. [22] prepared self-similar solutions for rotating viscoelastic fluid flow past an impermeable stretchable surface. Rosali et al. [23] numerically reported the rotating flow past an exponentially shrinking porous surface. Hayat et al. [24] utilized non-Fourier heat flux theory for three dimensional (3D) rotating flow of Jeffrey material. Mustafa et al. [25] examined nonlinear radiation characteristics in rotating nanofluid flow past a stretchable surface. Shafique et al. [26] studied rotating flow of Maxwell material with binary chemical reaction and activation energy. Hayat et al. [27] investigated three dimensional rotating flow of Maxwell nanofluid. Recently Hayat et al. [28] also provided an optimal study for three-dimensional flow of Maxwell nanofluid subject to rotating frame.

The investigators at present are involved in analyzing the fluid flow through porous space due to its abundant applications in hydrology, agriculture, civil engineering, petroleum technology, chemical engineering, nanofluid mechanics, mining and mineral processing and oil and gas production [29–33]. The available literature witnesses that classical Darcy's theory has been mostly used for modeling and analysis of flow problems of porous media. Note that classical Darcy's theory is valid for lower velocity and smaller porosity. Therefore Darcy's theory is inadequate when inertial and boundary effects take place at higher flow rate. Thus Forchheimer [34] analyzed such effects through square velocity factor in addition to Darcian velocity in momentum expression. Muskat [35] called this factor as "Forchheimer term". Seddeek [36] studied mixed convection flow in view of Darcy-Forchheimer relation. Jha et al. [37] developed nonlinear Brinkman-Forchheimer-extended Darcy flow model. Pal and Mondal [38] examined hydromagnetic Darcy-Forchheimer flow of variable viscosity liquid. Sadiq and Hayat [39] investigated Darcy-Forchheimer flow of magneto Maxwell liquid bounded by a convectively heated sheet. Cattaneo-Christov heat flux model in Darcy-Forchheimer flow of an Oldroyd-B fluid non-linear convection is studied by Shehzad et al. [40]. Bakar et al. [41] analyzed forced convection stagnation-point flow in a Darcy-Forchheimer porous medium towards a shrinking sheet. Hayat et al. [42] discussed Darcy-Forchheimer flow of Maxwell material subject to heat flux via Cattaneo-Christov theory and variable thermal conductivity. Umavathi et al. [43] performed numerical analysis of natural convective flow of nanofluids in a vertical rectangular duct using Darcy-Forchheimer-Brinkman model. Hayat et al. [44] provided a comparative study for Darcy-Forchheimer flow of viscoelastic nanofluids. Recently Hayat et al. [45] also discussed Darcy-Forchheimer flow of viscoelastic fluids with Cattaneo-Christov heat flux and homogeneous-heterogeneous reactions.

Motivated by above mentioned studies, our objective of present investigation are four folds. Firstly to model and analyze the Darcy-Forchheimer three-dimensional rotating flow of carbon nanotubes. Secondly to consider the rotating frame. Thirdly to use Xue model for nanofluid transport process. Fourth to construct convergent series solutions for velocities and temperature distributions using optimal homotopy analysis method (OHAM) [46–55]. The contributions of various flow variables are studied and discussed. Moreover skin-friction coefficients and heat transfer rate (local Nusselt number) are analyzed through numerical values.

## 2. Modeling

Here three-dimensional rotating flow of carbon nanotubes (CNTs) induced by a linearly stretchable surface is developed. Heat transfer aspects are studied through Xue model [3]. An incompressible liquid characterizing Darcy-Forchheimer relation saturates the porous space. Here we consider the Cartesian coordinate system such that the sheet is aligned with the *xy*− plane and fluid is considered for *z* ≥ 0. The surface is stretching in *x*− direction with rate *c* > 0. Moreover the fluid is subjected to uniform rotation about *z*− axis with constant angular velocity *ω*. The surface temperature is because of convective heating process which is featured by hot fluid temperature *T*_*f*_ and coefficient of heat transfer *h*_*f*_. The associated equations governing the Darcy-Forchheimer flow of carbon nanotubes (CNTs) in rotating frame are

$$\frac{\partial u}{\partial x}+\frac{\partial v}{\partial y}+\frac{\partial w}{\partial z}=0,$$ (1)

$$u\frac{\partial u}{\partial x}+v\frac{\partial u}{\partial y}+w\frac{\partial u}{\partial z}-2\omega v=\nu_{nf}\left(\frac{\partial^{2}u}{\partial z^{2}}\right)-\frac{\nu_{nf}}{K^{*}}u-Fu^{2},$$ (2)

$$u\frac{\partial v}{\partial x}+v\frac{\partial v}{\partial y}+w\frac{\partial v}{\partial z}+2\omega u=\nu_{nf}\left(\frac{\partial^{2}v}{\partial z^{2}}\right)-\frac{\nu_{nf}}{K^{*}}v-Fv^{2},$$ (3)

$$u\frac{\partial T}{\partial z}+v\frac{\partial T}{\partial y}+w\frac{\partial T}{\partial z}=\alpha_{nf}\frac{\partial^{2}T}{\partial z^{2}},$$ (4)

with boundary conditions are

$$u=u_{w}\left(x\right)=cx,\;v=0,\;w=0,\;-k_{nf}\frac{\partial T}{\partial z}=h_{f}\left(T_{f}-T\right)\text{ at }z=0,$$ (5)

$$u\to 0,\;v\to 0,\;T\to T_{\infty }\text{ as }z\to \infty .$$ (6)

Here *u*, *v* and *w* are the components of velocity in *x*−, *y*− and *z*− directions respectively, K* is the permeability of porous medium, $F=\frac{C_{b}}{xK^{{*}^{1/2}}}$ the non-uniform inertia coefficient of porous medium, *C*_*b*_ the drag coefficient, *T* the temperature and *T*_∞_ the ambient fluid temperature. The theoretical model proposed by Xue [3] is presented as follows:

$$\begin{array}{ll} \mu_{nf} & =\frac{\mu_{f}}{{\left(1-\phi \right)}^{2.5}},\;\nu_{nf}=\frac{\mu_{nf}}{\rho_{nf}},\;\alpha_{nf}=\frac{k_{nf}}{{\left(\rho c_{p}\right)}_{nf}},\;\rho_{nf}=\rho_{f}\left(1-\phi \right)+\rho_{CNT}\phi ,\\ {\left(\rho c_{p}\right)}_{nf} & ={\left(\rho c_{p}\right)}_{f}\left(1-\phi \right)+{\left(\rho c_{p}\right)}_{CNT}\phi ,\;\frac{k_{nf}}{k_{f}}=\frac{\left(1-\phi \right)+2\phi \frac{k_{CNT}}{k_{CNT}-k_{f}}\text{ln}\frac{k_{CNT}+k_{f}}{2k_{f}}}{\left(1-\phi \right)+2\phi \frac{k_{f}}{k_{CNT}-k_{f}}\text{ln}\frac{k_{CNT}+k_{f}}{2k_{f}}}, \end{array}$$ (7)

where *ϕ* is the solid volume fraction of nanoparticles, *μ*_*nf*_ the nanofluid effective dynamic viscosity of nanofluids, *ρ*_*nf*_ the density of nanofluids, (*ρc*_*P*_)_*nf*_ the heat capacity of nanofluids, *k*_*nf*_ the thermal conductivity of nanofluid, *ρ*_*CNT*_ the density of carbon nanotubes, *ρ*_*f*_ the density of base fluid, *k*_*CNT*_ the thermal conductivity of CNTs and *k*_*f*_ the thermal conductivity of base fluid. Table 1 presents the thermophysical characteristics of water and CNTs.

**Table 1:** Thermophysical characteristics of water and carbon nanotubes [3].

Physical properties	Water	Nanoparticles	
		SWCNTs	MWCNTs
*ρ*(*kg*/*m*^3^)	997.1	2600	1600
*k*(*W*/*mK*)	0.613	6600	3000
*c*_*p*_(*J*/*kgK*)	4179	425	796

Considering [28]:

$$\begin{array}{c} u=cxf^{\prime }(\zeta ),\;v=cxg\left(\zeta \right),\;w=-{\left(c\nu_{f}\right)}^{1/2}f(\zeta ),\\ \theta (\zeta )=\frac{T-T_{\infty }}{T_{f}-T_{\infty }},\;\zeta ={\left(\frac{c}{\nu_{f}}\right)}^{1/2}z. \end{array}$$ (8)

Now Eq (1) is identically verified and Eqs (2)–(7) have been reduced to

$$\frac{1}{{\left(1-\phi \right)}^{2.5}\left(1-\phi +\frac{\rho_{CNT}}{\rho_{f}}\phi \right)}\left(f^{\prime \prime \prime }-\lambda f^{\prime }\right)+ff^{\prime \prime }+2\Omega g-\left(1+F_{r}\right)\;{f^{\prime }}^{{}^{2}}=0,$$ (9)

$$\frac{1}{{\left(1-\phi \right)}^{2.5}\left(1-\phi +\frac{\rho_{CNT}}{\rho_{f}}\phi \right)}\left(g^{\prime \prime }-\lambda g\right)+fg^{\prime }-f^{\prime }g-2\Omega f^{\prime }-F_{r}g^{2}=0,$$ (10)

$$\frac{1}{\left(1-\phi +\frac{{\left(\rho c_{p}\right)}_{CNT}}{{\left(\rho c_{p}\right)}_{f}}\phi \right)}\frac{k_{nf}}{k_{f}}\theta^{\prime \prime }+\text{ Pr }f\theta^{\prime }=0,$$ (11)

$$f=0,\;f^{\prime }=1,\;g=0,\;\theta^{\prime }=-\frac{k_{f}}{k_{nf}}\gamma \left(1-\theta \right)\text{ at }\zeta =0,$$ (12)

$$f^{\prime }\to 0,\;g\to 0,\;\theta \to 0\text{ when }\zeta \to \infty .$$ (13)

Here *λ* is the porosity parameter, Ω the rotation parameter, *F*_*r*_ the inertia coefficient, Pr the Prandtl number and *γ* Biot number. These parameters can be specified by using the definitions given below:

$$\lambda =\frac{\nu_{f}}{cK^{*}},\;F_{r}=\frac{C_{b}}{K^{{*}^{1/2}}},\;\Omega =\frac{\omega }{c},\text{ Pr }=\frac{\nu_{f}}{\alpha_{f}},\;\gamma =\frac{h_{f}}{k_{f}}\sqrt{\frac{\nu_{f}}{c}}.$$ (14)

Skin-friction coefficients and local Nusselt number are given by

$$\begin{array}{l} C_{fx}\text{R}{\text{e}}_{x}^{1/2}=\frac{1}{{\left(1-\phi \right)}^{2.5}}f^{\prime \prime }\left(0\right),\\ C_{fy}\text{R}{\text{e}}_{x}^{1/2}=\frac{1}{{\left(1-\phi \right)}^{2.5}}g^{\prime }\left(0\right),\\ Nu_{x}\text{R}{\text{e}}_{x}^{-1/2}=-\frac{k_{nf}}{k_{f}}\theta^{\prime }\left(0\right), \end{array}\}\;$$ (15)

in which $\text{R}{\text{e}}_{x}=\frac{u_{w}x}{\nu }$ depicts the local Reynolds number. It is noticed that the present analysis is reduced to conventional fluid case when *ϕ* = 0.

## 3. Solutions by OHAM

The series solutions of Eqs (9)–(11) through Eqs (12) and (13) have been constructed by utilizing optimal homotopy analysis method (OHAM). The initial approximations and linear operators have been selected as follows:

$$f_{0}(\zeta )=1-e^{-\zeta },\;g_{0}\left(\zeta \right)=0,\;\theta_{0}(\zeta )=\frac{\gamma }{\gamma +\frac{k_{nf}}{k_{f}}}e^{-\zeta },$$ (16)

$${\text{L}}_{f}=\frac{d^{3}f}{d\zeta^{3}}-\frac{df}{d\zeta },\;{\text{L}}_{g}=\frac{d^{2}g}{d\zeta^{2}}-g,\;{\text{L}}_{\theta }=\frac{d^{2}\theta }{d\zeta^{2}}-\theta .$$ (17)

The above linear operators obey

$${\text{L}}_{f}\left[F_{1}^{**}+F_{2}^{**}e^{\zeta }+F_{3}^{**}e^{-\zeta }\right]=0,\;{\text{L}}_{g}\left[F_{4}^{**}e^{\zeta }+F_{5}^{**}e^{-\zeta }\right]=0,\;{\text{L}}_{\theta }\left[F_{6}^{**}e^{\zeta }+F_{7}^{**}e^{-\zeta }\right]=0,\;$$ (18)

in which $F_{j}^{*}(j=1-7)$ depict the arbitrary constants. The zeroth and mth-order deformation problems can be easily developed in view of above linear operators. The deformation problems have been computed through BVPh 2.0 of software Mathematica.

## 4. Optimal convergence control parameters

The non-zero auxiliary parameters *ℏ*_*f*_, *ℏ*_*g*_ and *ℏ*_*θ*_ in homotopy solutions regulate the convergence region and also rate of homotopy expressions. To obtain the optimal data of *ℏ*_*f*_, *ℏ*_*g*_ and *ℏ*_*θ*_, we have used the idea of minimization by defining the average squared residual errors as proposed by Liao [46].

$$\varepsilon_{m}^{f}=\frac{1}{k+1}\overset{k}{\underset{j=0}{\sum }}{\left[{\text{N}}_{f}{\left(\overset{m}{\underset{i=0}{\sum }}\hat{f}\left(\zeta \right),\;\overset{m}{\underset{i=0}{\sum }}\hat{g}\left(\zeta \right)\right)}_{\zeta =j\delta \zeta }\right]}^{2},$$ (19)

$$\varepsilon_{m}^{g}=\frac{1}{k+1}\overset{k}{\underset{j=0}{\sum }}{\left[{\text{N}}_{g}{\left(\overset{m}{\underset{i=0}{\sum }}\hat{f}\left(\zeta \right),\;\overset{m}{\underset{i=0}{\sum }}\hat{g}\left(\zeta \right)\right)}_{\zeta =j\delta \zeta }\right]}^{2},$$ (20)

$$\varepsilon_{m}^{\theta }=\frac{1}{k+1}\overset{k}{\underset{j=0}{\sum }}{\left[{\text{N}}_{\theta }{\left(\overset{m}{\underset{i=0}{\sum }}\hat{f}\left(\zeta \right),\;\overset{m}{\underset{i=0}{\sum }}\hat{g}\left(\zeta \right),\;\overset{m}{\underset{i=0}{\sum }}\hat{\theta }\left(\zeta \right)\right)}_{\zeta =j\delta \zeta }\right]}^{2}.$$ (21)

**Fig 1:**
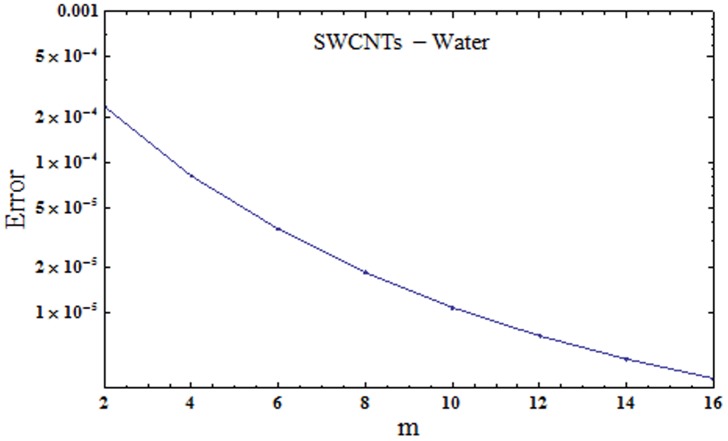
Total residual error for SWCNTs.

**Fig 2:**
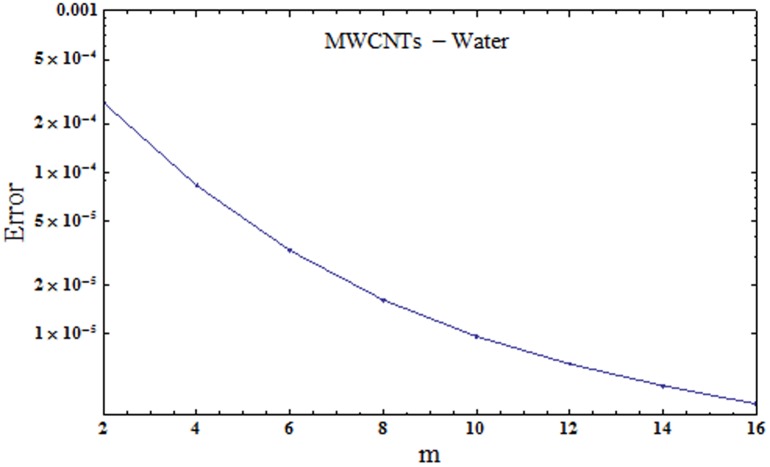
Total residual error for MWCNTs.

Following Liao [46]:

$$\varepsilon_{m}^{t}=\varepsilon_{m}^{f}+\varepsilon_{m}^{g}+\varepsilon_{m}^{\theta },$$ (22)

where $\varepsilon_{m}^{t}$ stands for total squared residual error, *δζ* = 0.5 and *k* = 20. The optimal data of convergence control parameters at 2nd order of approximations for SWCNTs are *ℏ*_*f*_ = -1.162, *ℏ*_*g*_ = -1.24942 and *ℏ*_*θ*_ = -0.454802 and the total averaged squared residual error is $\varepsilon_{m}^{t}=2.36\times 10^{-4}$ while the optimal data of convergence control parameters at 2nd order of approximations for MWCNTs are *ℏ*_*f*_ = -1.03993, *ℏ*_*g*_ = -1.15222 and *ℏ*_*θ*_ = -0.510592 and total averaged squared residual error is $\varepsilon_{m}^{t}=2.70\times 10^{-4}$. Figs 1 and 2 demonstrate total residual error plots for SWCNTs and MWCNTs respectively. Tables 2 and 3 present individual average squared residual errors considering the optimal data of convergence control variables at *m* = 2. It has been found that averaged squared residual error reduces with higher order deformations.

**Table 2:** Individual averaged squared residual errors for SWCNTs considering optimal data of auxiliary parameters.

*m*	$\varepsilon_{m}^{f}$	$\varepsilon_{m}^{\text{g}}$	$\varepsilon_{m}^{\theta }$
2	2.92 × 10^−5^	9.26 × 10^−5^	1.14× 10^−4^
6	4.68 × 10^−6^	1.43× 10^−5^	1.71× 10^−5^
10	2.12 × 10^−6^	5.55× 10^−6^	3.17× 10^−6^
14	1.26 × 10^−6^	2.87× 10^−6^	8.06× 10^−7^
16	1.02 × 10^−6^	2.19× 10^−6^	4.53× 10^−7^

**Table 3:** Individual averaged squared residual errors for MWCNTs considering optimal data of auxiliary parameters.

*m*	$\varepsilon_{m}^{f}$	$\varepsilon_{m}^{\text{g}}$	$\varepsilon_{m}^{\theta }$
2	6.59 × 10^−6^	1.07 × 10^−4^	1.56× 10^−4^
6	1.28 × 10^−6^	1.84× 10^−5^	1.32× 10^−5^
10	6.85 × 10^−7^	7.60× 10^−6^	1.34× 10^−6^
14	4.55 × 10^−7^	4.12× 10^−6^	1.95× 10^−7^
16	3.86 × 10^−7^	3.21× 10^−6^	8.46× 10^−8^

## 5. Discussion

**Fig 3:**
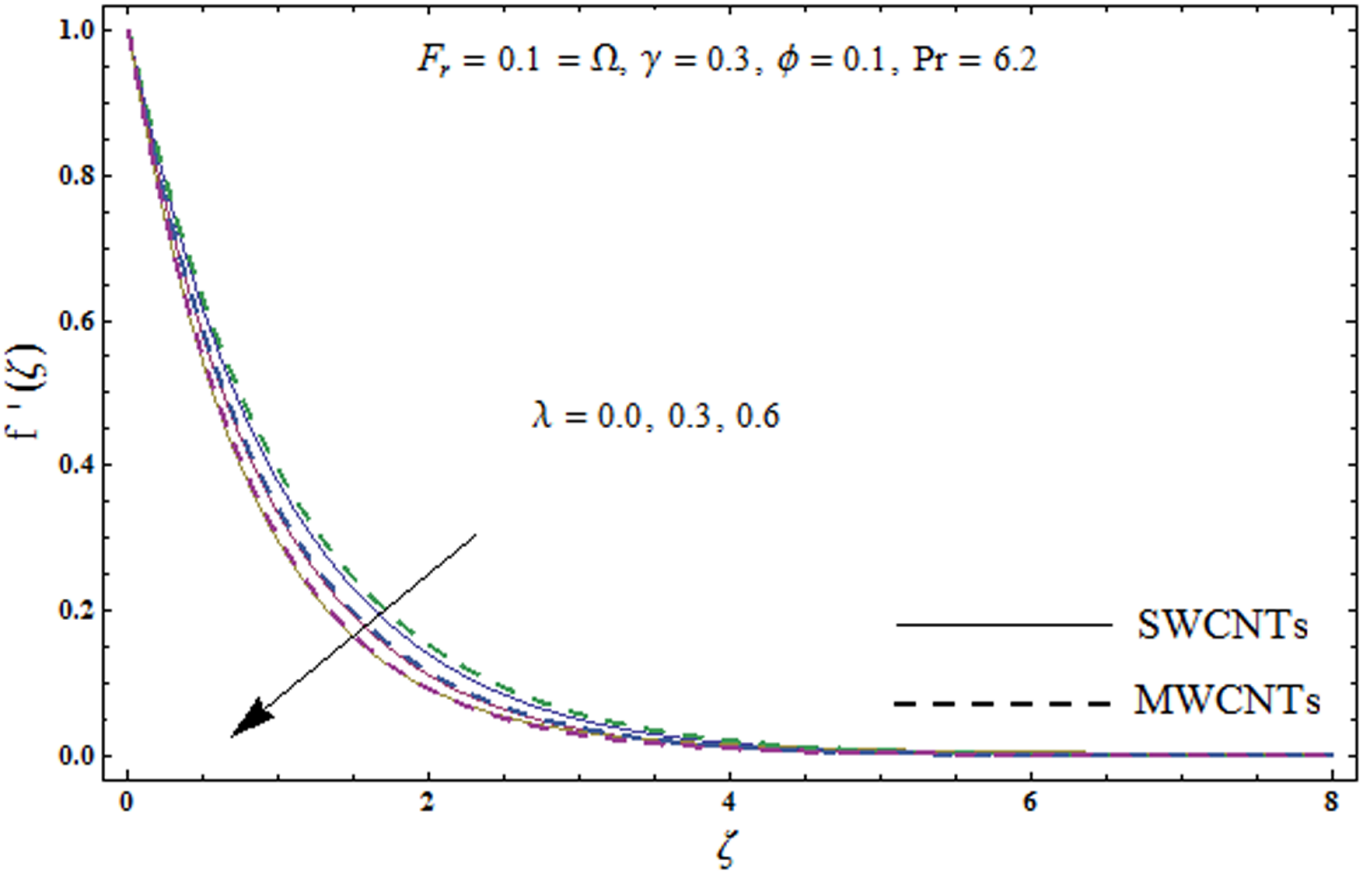
Plots of velocity field *f*′(*ζ*) for porosity parameter *λ*.

**Fig 4:**
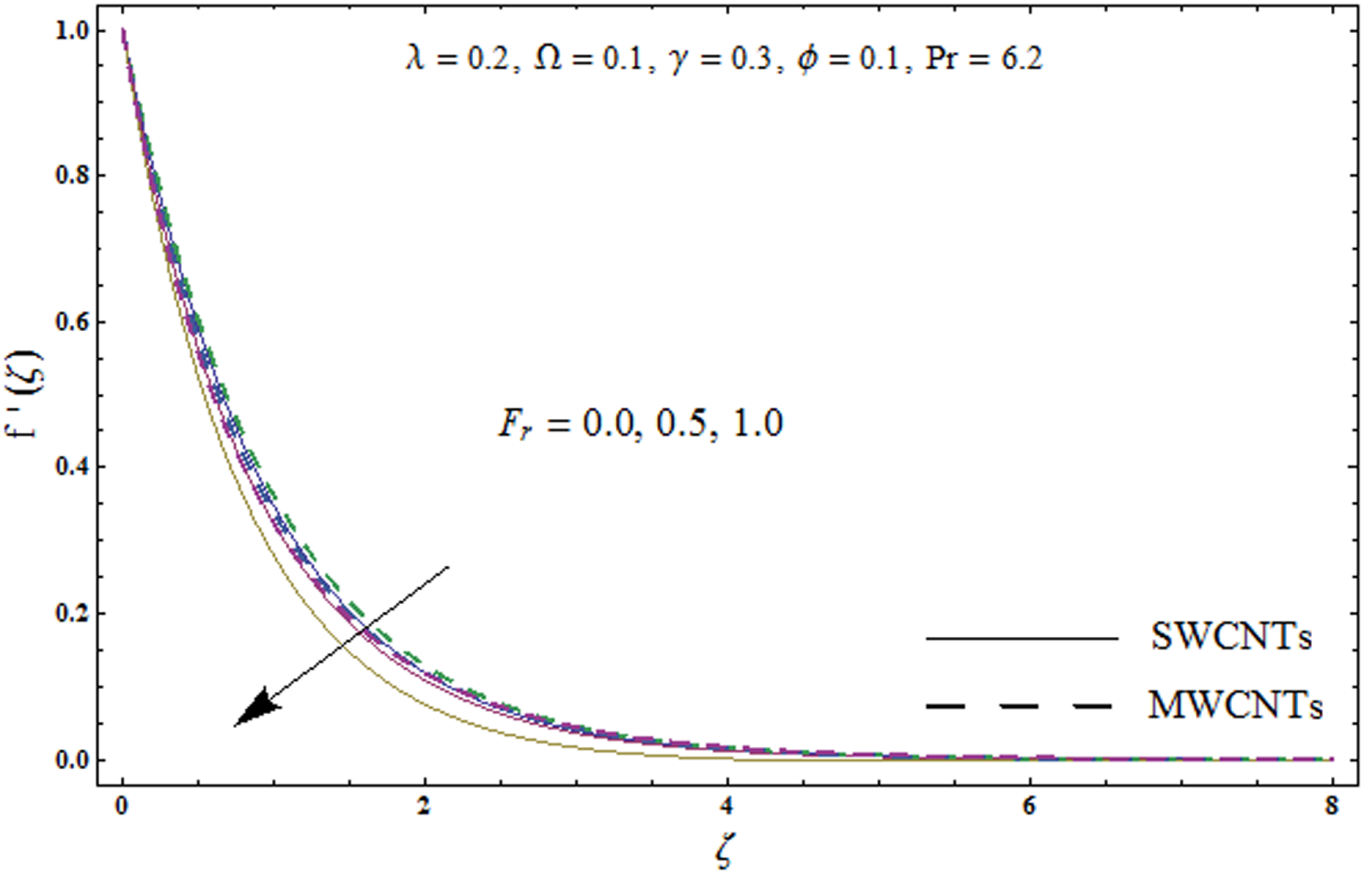
Plots of velocity field *f*′(*ζ*) for inertia coefficient *F*_*r*_.

**Fig 5:**
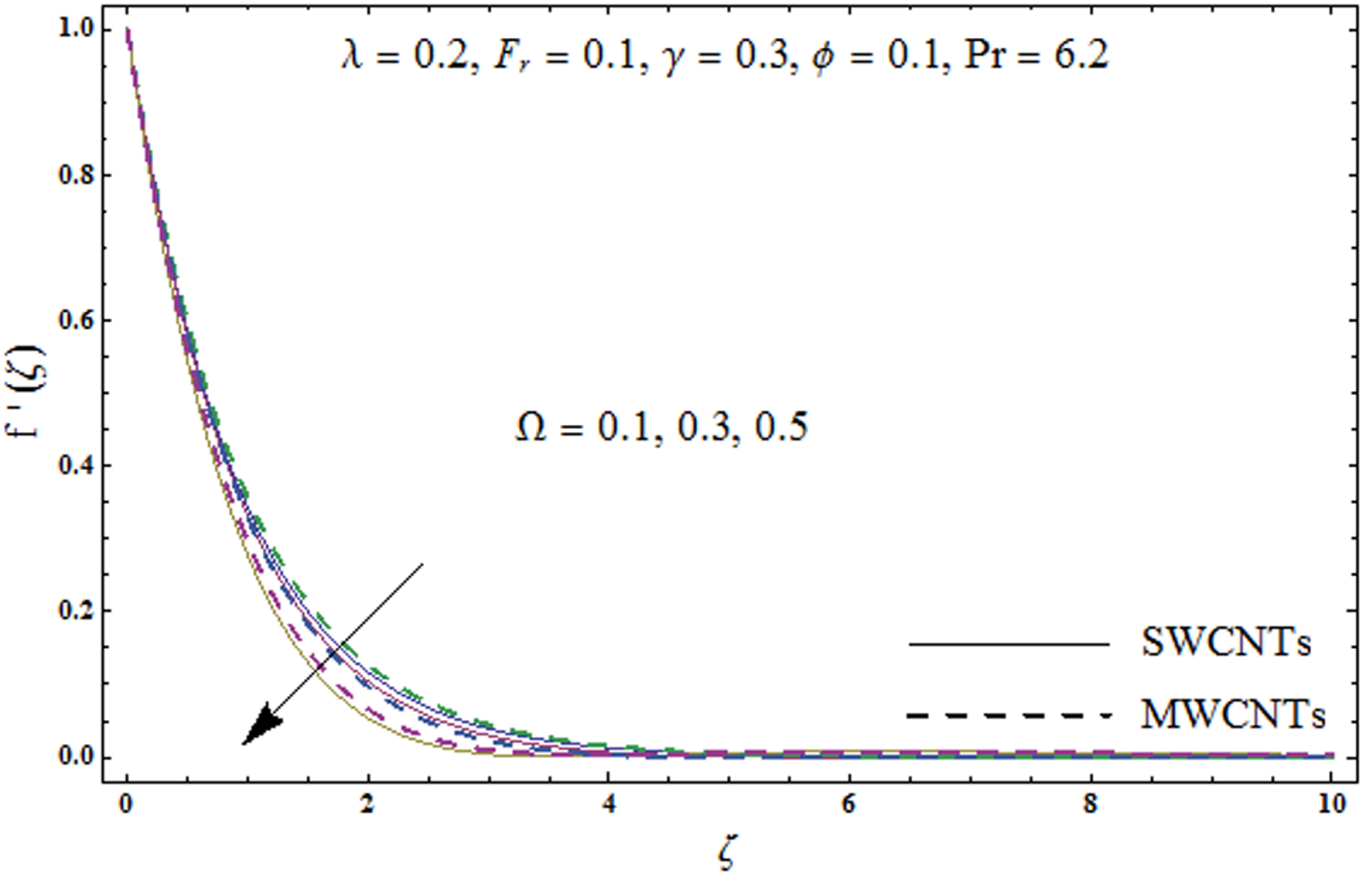
Plots of velocity field *f*′(*ζ*) for rotation parameter *Ω*.

**Fig 6:**
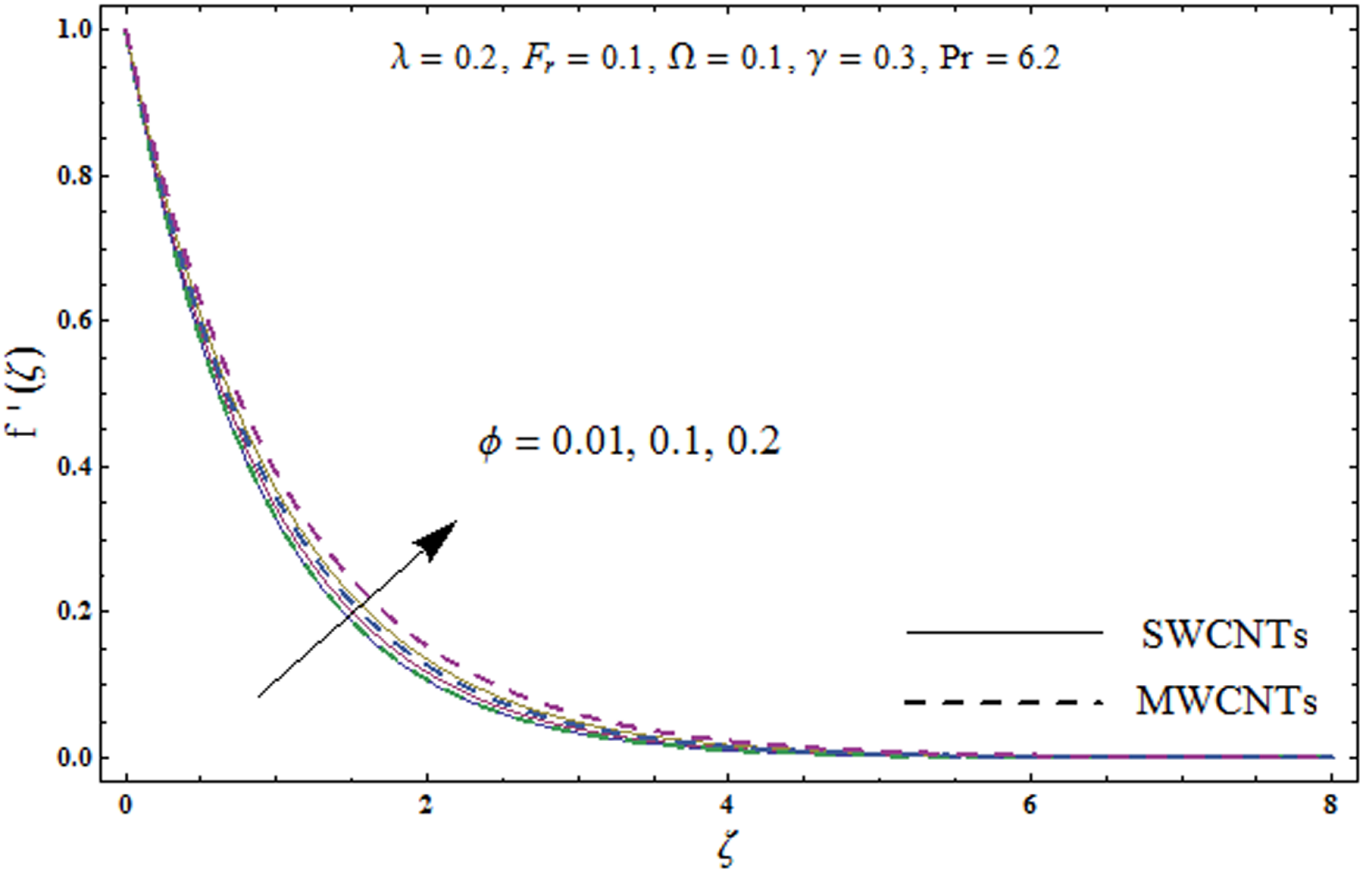
Plots of velocity field *f*′(*ζ*) for nanoparticles volume fraction *ϕ*.

**Fig 7:**
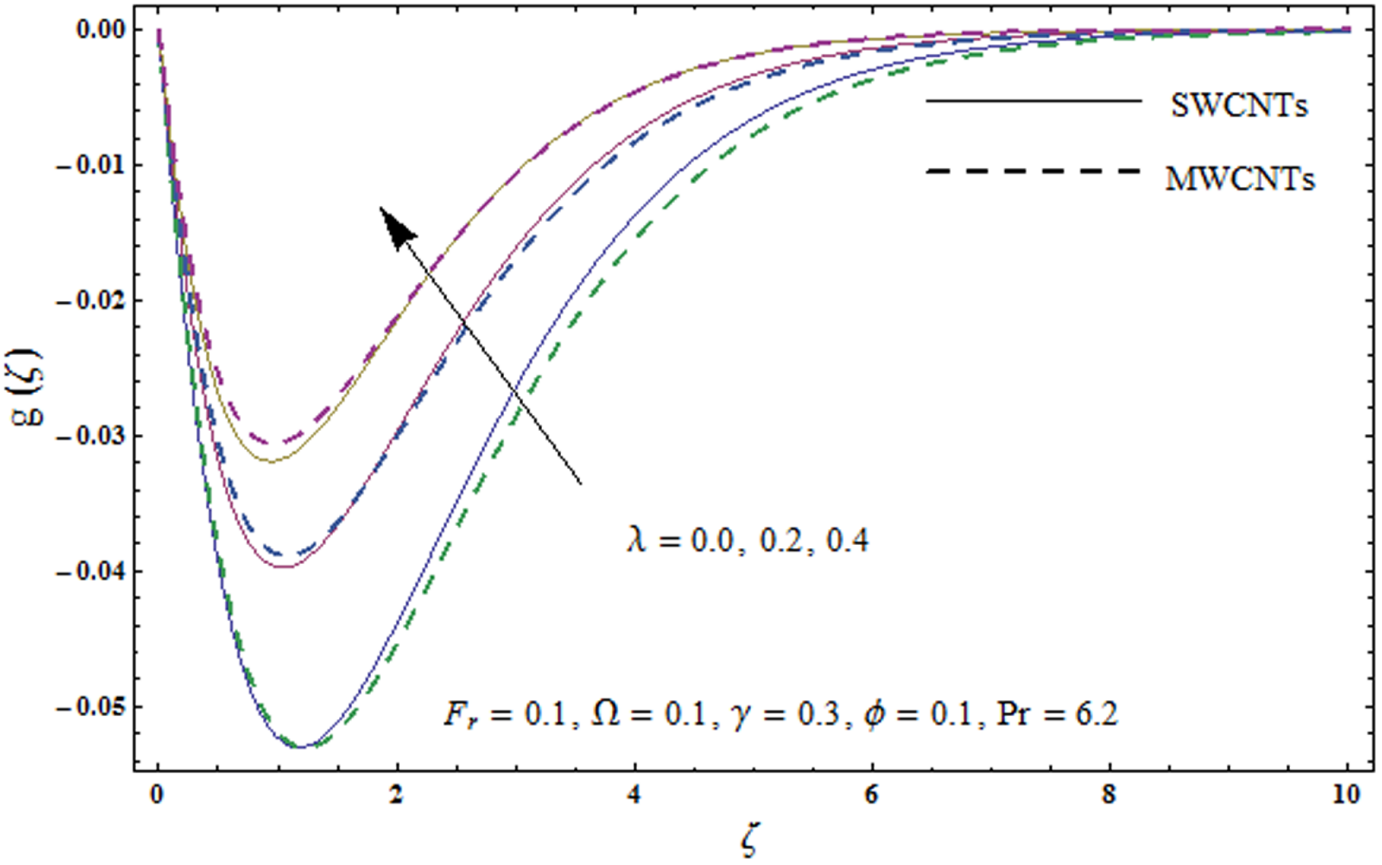
Plots of velocity field g(*ζ*) for porosity parameter *λ*.

**Fig 8:**
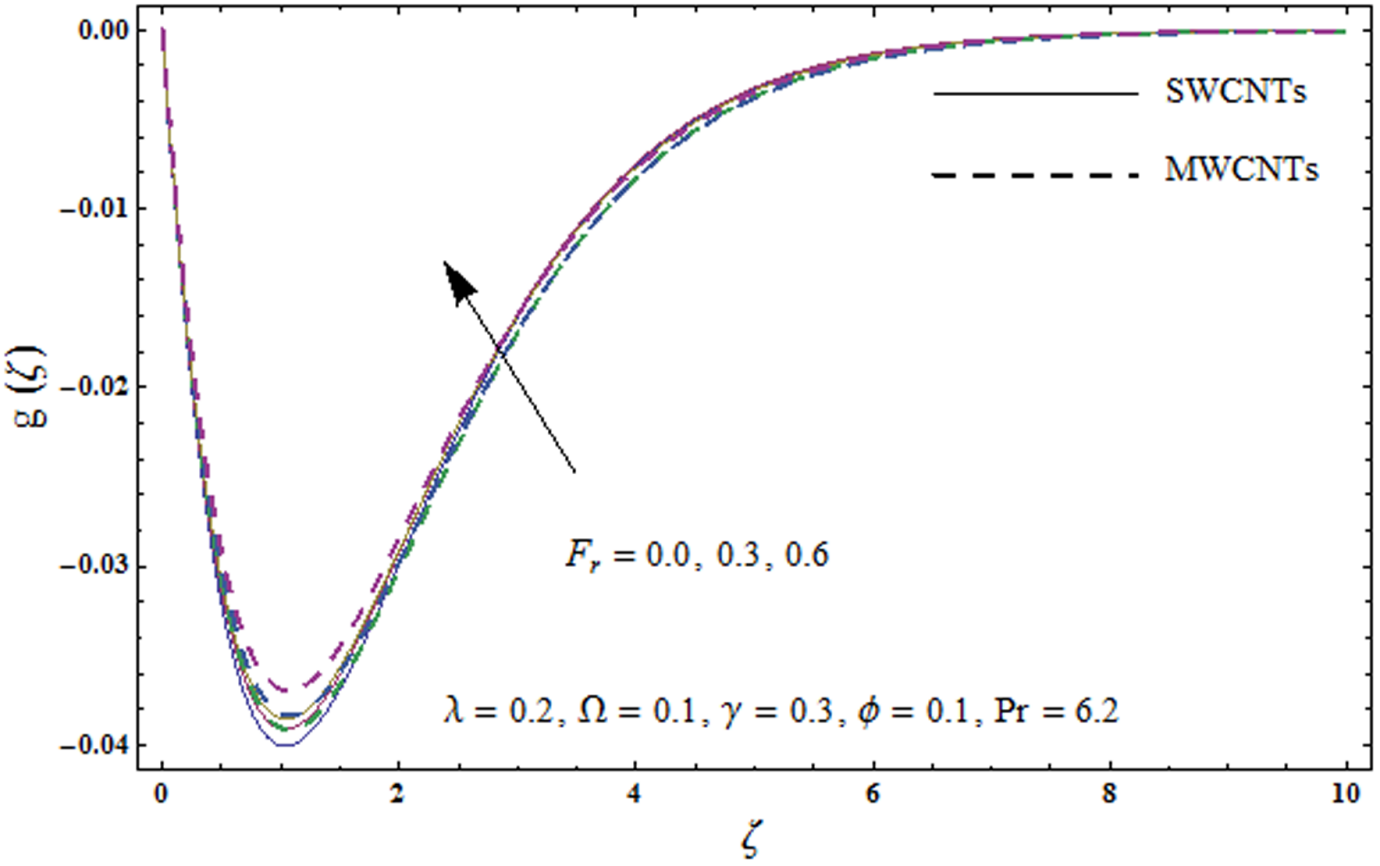
Plots of velocity field g(*ζ*) for inertia coefficient *F*_*r*_.

**Fig 9:**
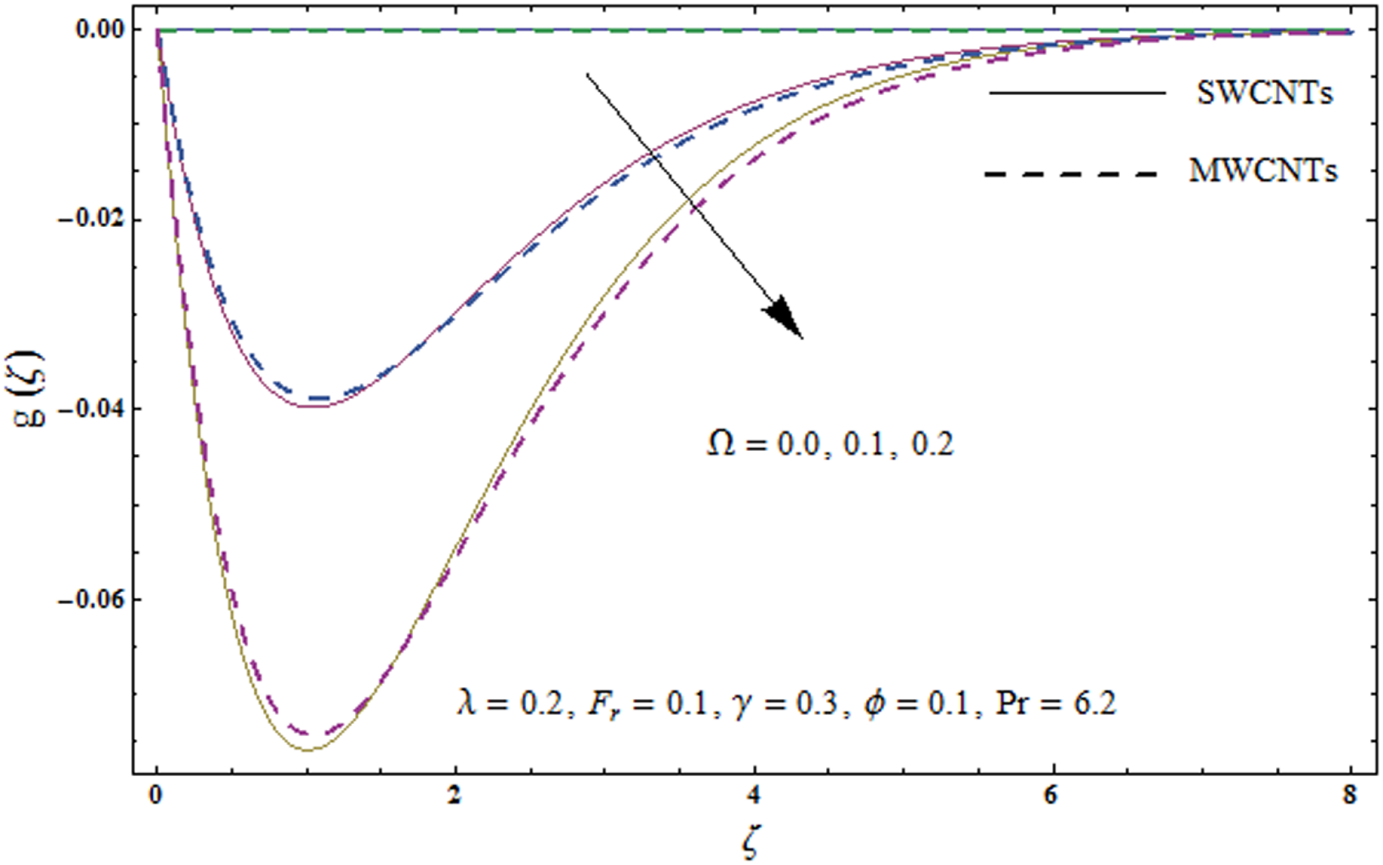
Plots of velocity field g(*ζ*) for rotation parameter *Ω*.

**Fig 10:**
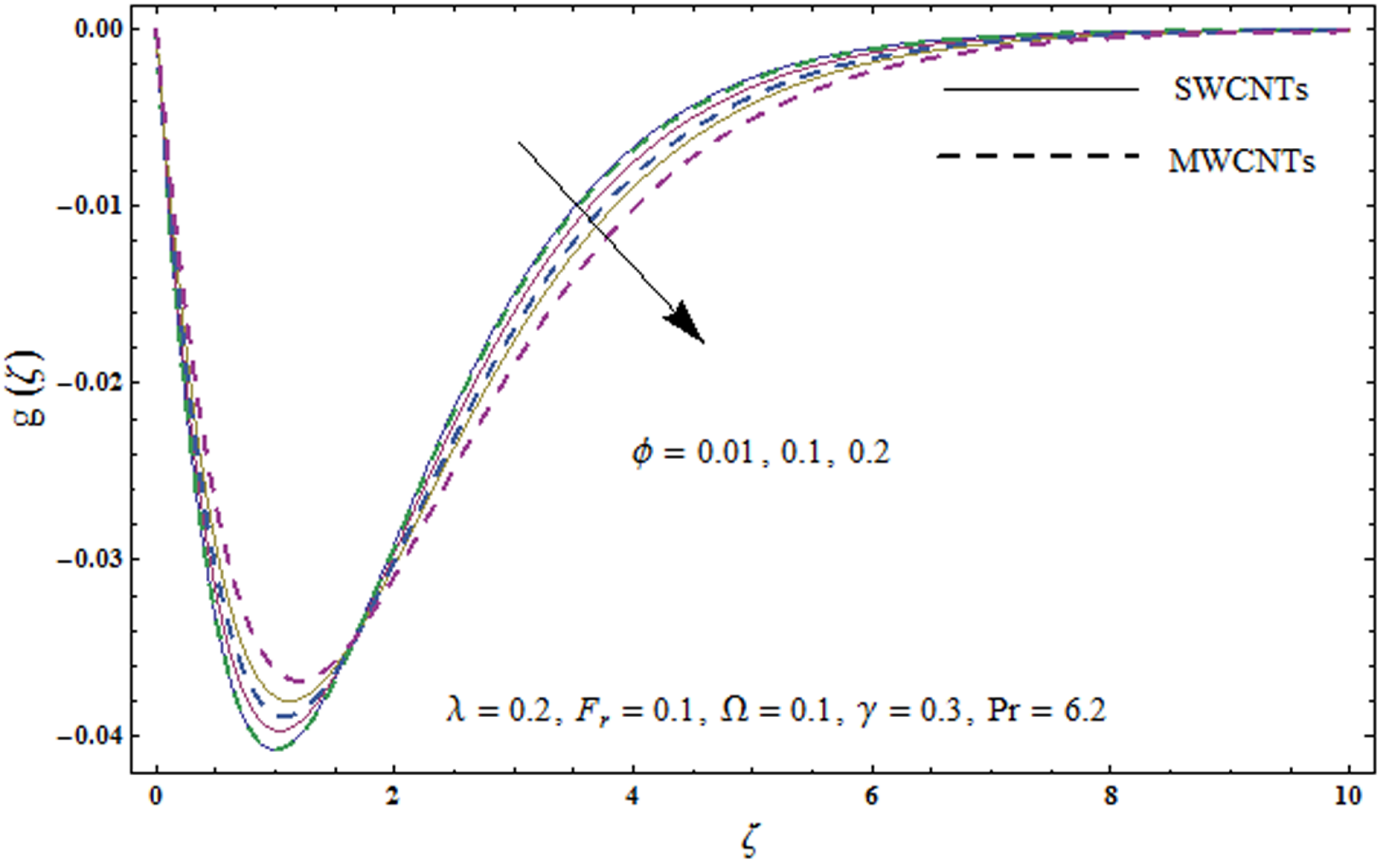
Plots of velocity field g(*ζ*) for nanoparticles volume fraction *ϕ*.

**Fig 11:**
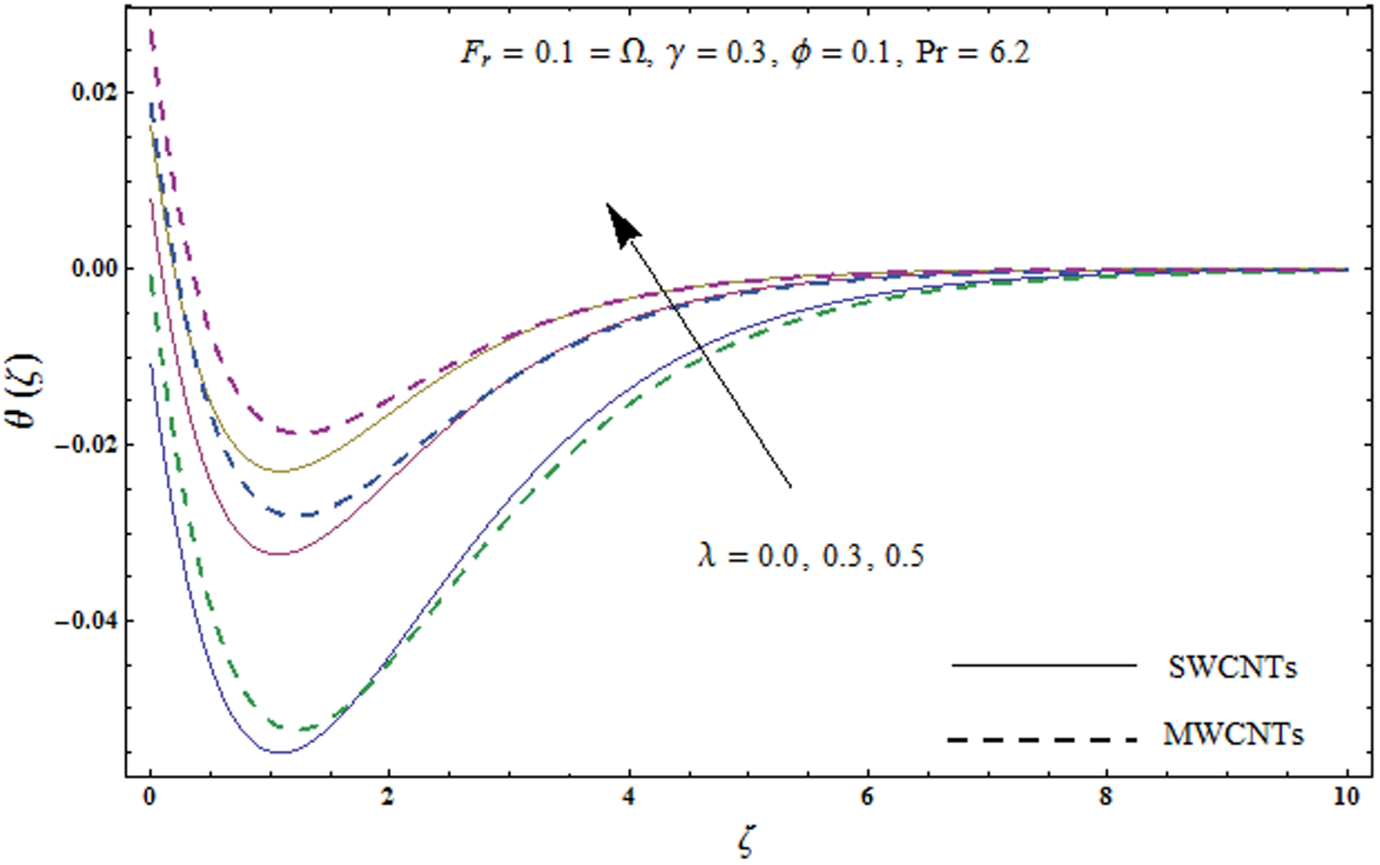
Plots of temperature field *θ*(*ζ*) for porosity parameter *λ*.

**Fig 12:**
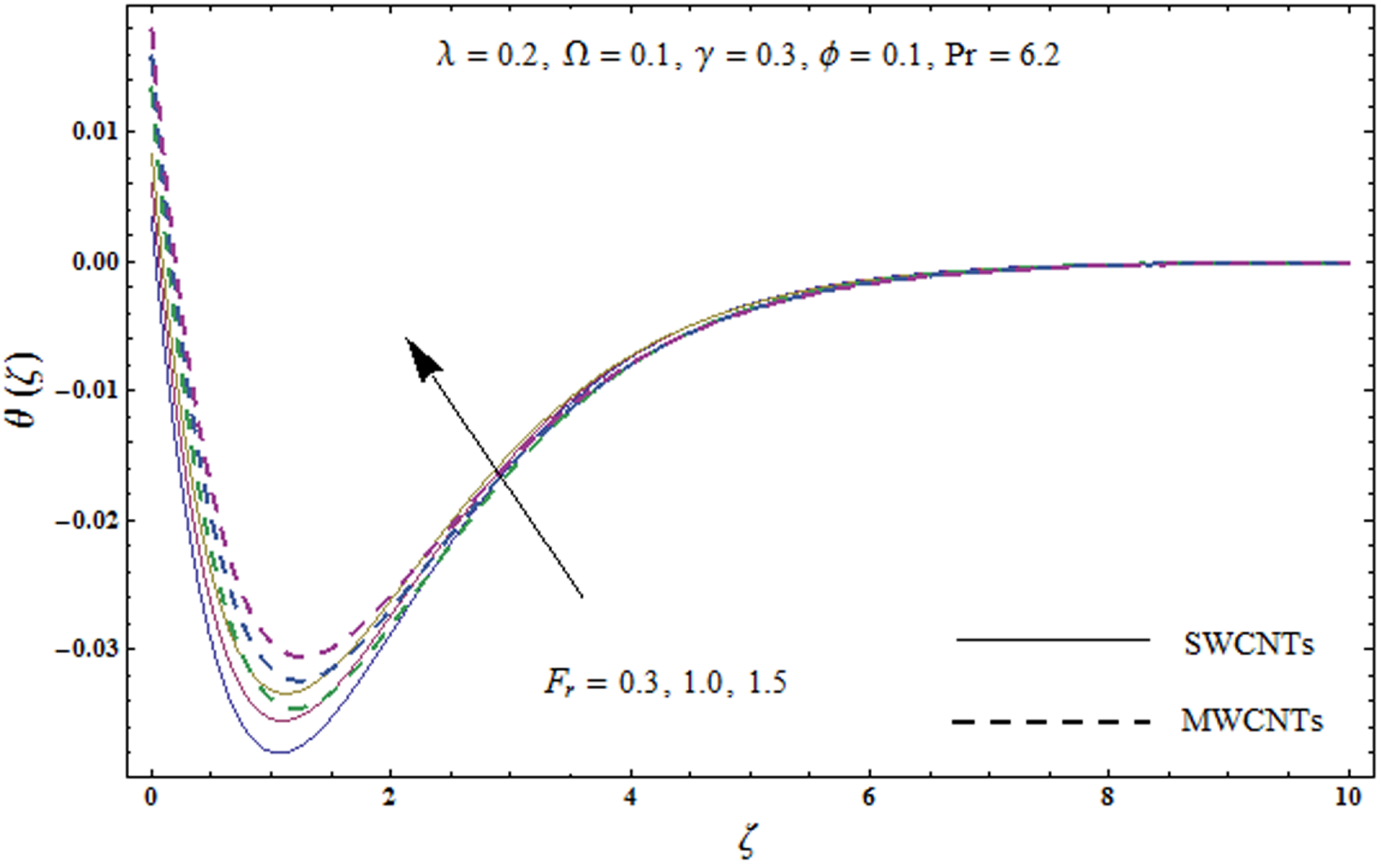
Plots of temperature field *θ*(*ζ*) for inertia coefficient *F*_*r*_.

**Fig 13:**
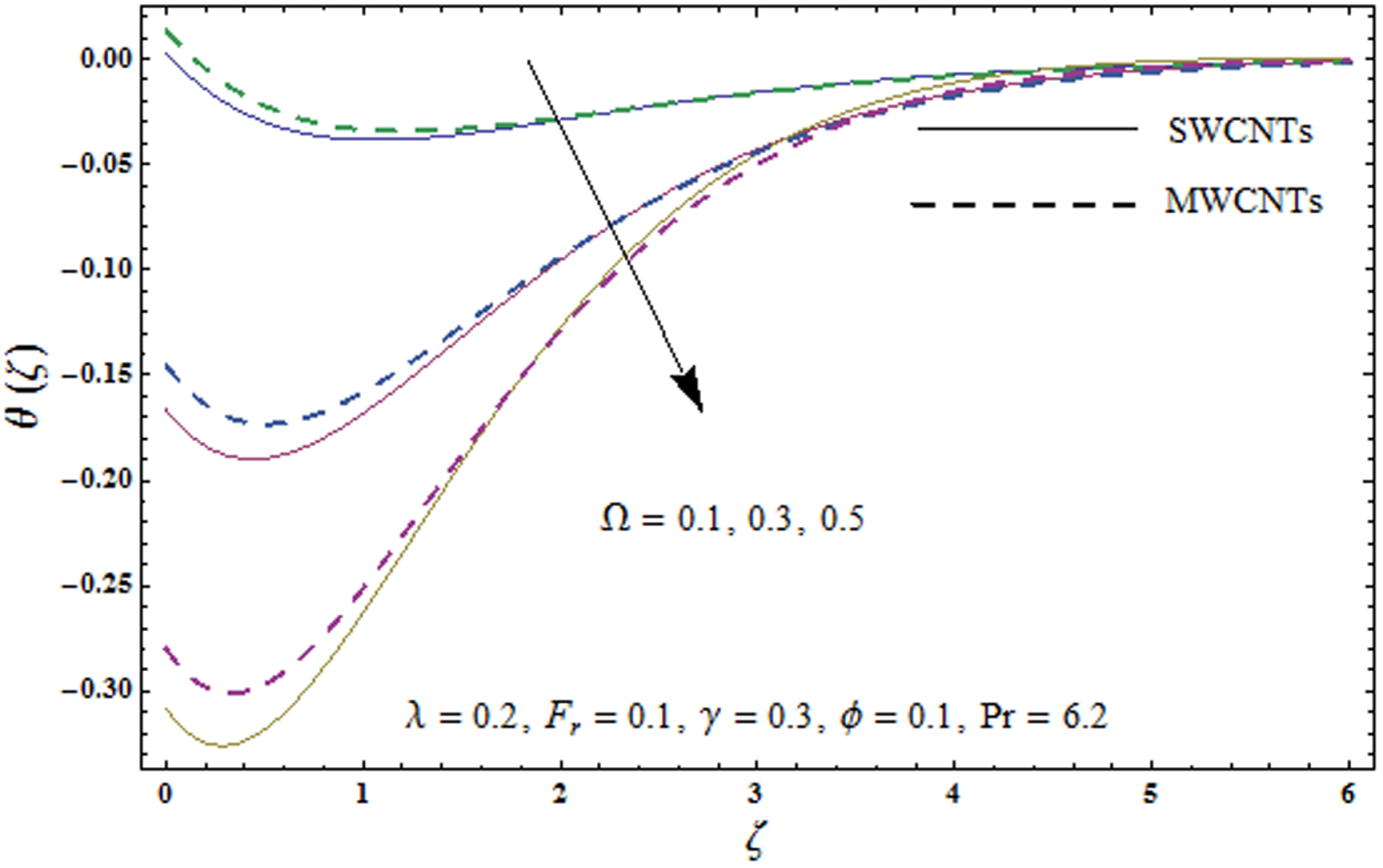
Plots of temperature field *θ*(*ζ*) for rotation parameter *Ω*.

**Fig 14:**
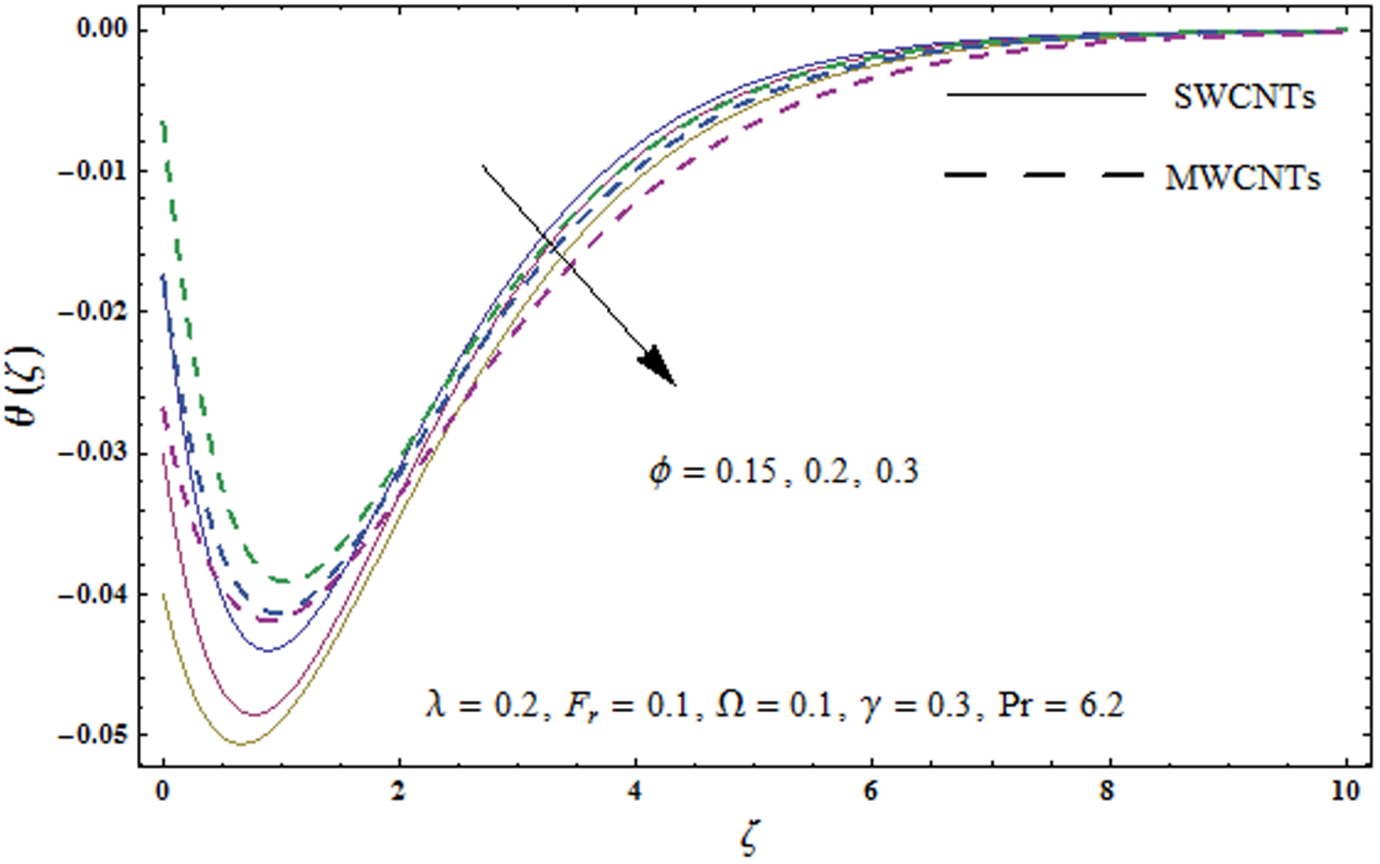
Plots of temperature field *θ*(*ζ*) for nanoparticles volume fraction *ϕ*.

**Fig 15:**
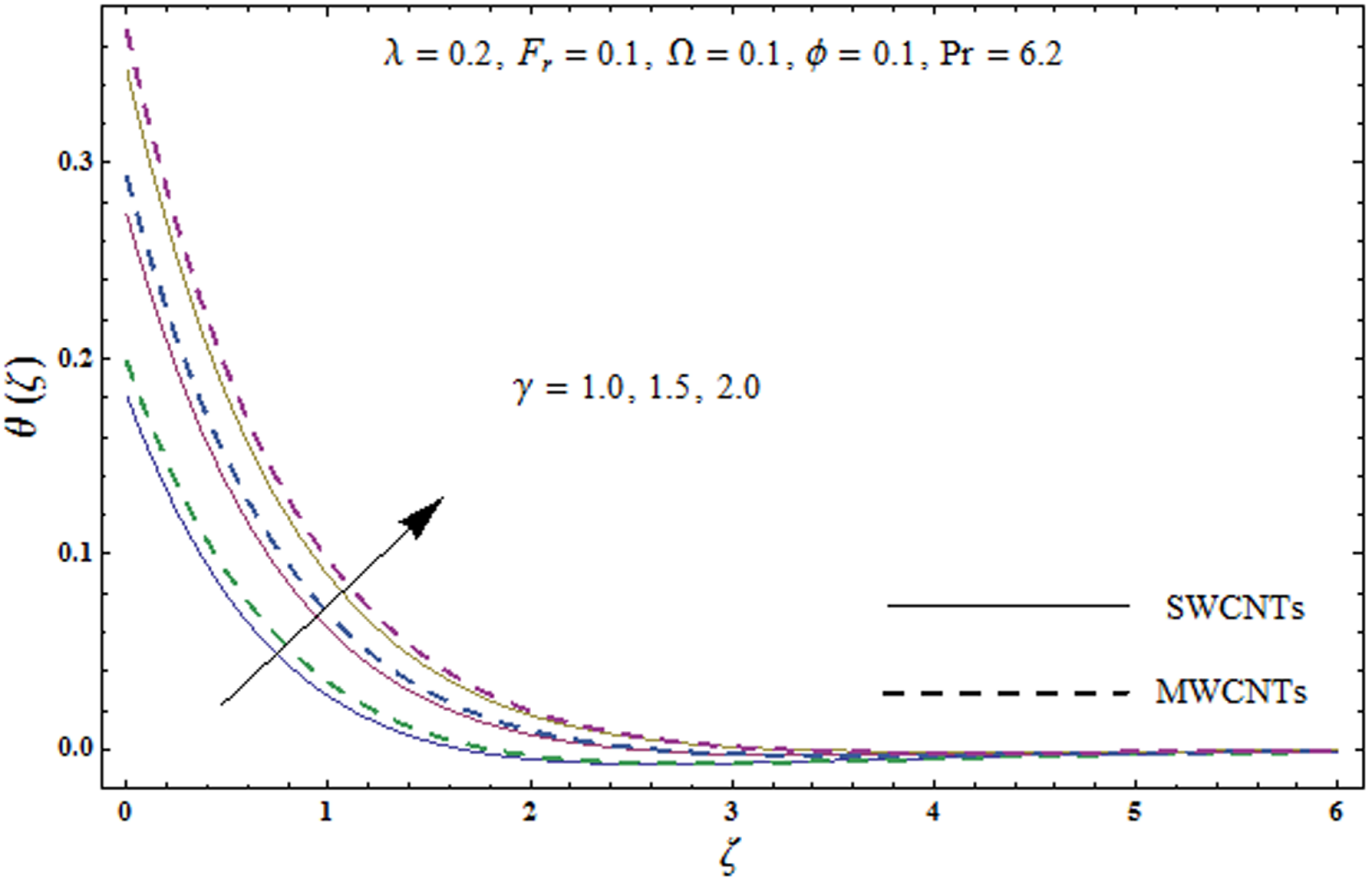
Plots of temperature field *θ*(*ζ*) for Biot number *γ*.

This portion explores the effects of various flow variables like porosity parameter *λ*, inertia coefficient *F*_*r*_, rotation parameter Ω, nanoparticles volume fraction *ϕ* and Biot number *γ* on the velocities *f*′(*ζ*) and *g*(*ζ*) and temperature *θ*(*ζ*) distributions. The results are achieved for both single wall carbon nanotubes (SWCNTs) and multi wall carbon nanotubes (MWCNTs). Fig 3 shows the variation in velocity field *f*′(*ζ*) for varying porosity parameter *λ*. It has been noticed that higher values of porosity parameter *λ* shows reduction in the velocity field *f*′(*ζ*) for both SWCNTs and MWCNTs. Fig 4 presents that higher values of inertia coefficient *F*_*r*_ causes a decay in velocity field *f*′(*ζ*) for both SWCNTs and MWCNTs. Fig 5 illustrates that how the rotation parameter Ω affects the velocity field *f*′(*ζ*). An increase in the values of rotation parameter Ω produces lower velocity field *f*′(*ζ*) and less momentum layer thickness for both SWCNTs and MWCNTs. Larger values of rotation parameter Ω leads to higher rotation rate in comparison to stretching rate. Therefore the larger rotation effects correspond to lower velocity field *f*′(*ζ*) and less momentum layer thickness. Fig 6 shows the variation in the velocity field *f*′(*ζ*) for varying nanoparticles volume fraction *ϕ*. It has been noticed that by increasing nanoparticles volume fraction *ϕ*, an enhancement in velocity field *f*′(*ζ*) is observed for both SWCNTs and MWCNTs. Fig 7 plots the velocity field *g*(*ζ*) for varying porosity parameter *λ*. It has been observed that velocity field *g*(*ζ*) is reduced for higher values of porosity parameter *λ* for both SWCNTs and MWCNTs. Fig 8 shows that how the inertia coefficient *F*_*r*_ affects the velocity field *g*(*ζ*). An enhancement in the velocity field *g*(*ζ*) is noticed for higher inertia coefficient for both SWCNTs and MWCNTs. Fig 9 plots the velocity field *g*(*ζ*) for varying rotation parameter Ω. It has been seen that rotation parameter has a vital role in accelerating the flow along the *y*− direction. Higher values of rotation parameter Ω correspond to an oscillatory trend in the velocity field *g*(*ζ*) for both SWCNTs and MWCNTs. Effects of nanoparticles volume fraction *ϕ* on the velocity field *g*(*ζ*) is presented in Fig 10. Larger nanoparticle volume fraction *ϕ* leads to an enhancement in the velocity field *g*(*ζ*) for both SWCNTs and MWCNTs. Fig 11 depicts the effect of porosity parameter *λ* on the temperature field *θ*(*ζ*). It has been noticed that by increasing porosity parameter *λ*, an enhancement in the temperature field *θ*(*ζ*) is observed for both SWCNTs and MWCNTs. Effects of inertia coefficient *F*_*r*_ on the temperature field *θ*(*ζ*) is sketched in Fig 12. Higher values of inertia coefficient *F*_*r*_ leads to stronger temperature field *θ*(*ζ*) and more thermal layer thickness for both SWCNTs and MWCNTs. Fig 13 demonstrates that how the temperature field *θ*(*ζ*) is affected by varying rotation parameter Ω. Larger rotation parameter Ω corresponds to stronger temperature field *θ*(*ζ*) and more thermal layer thickness for both SWCNTs and MWCNTs. Fig 14 plots the temperature field *θ*(*ζ*) for varying nanoparticles volume fraction *ϕ* for both SWCNTs and MWCNTs. It has been found that larger nanoparticles volume fraction *ϕ* leads to stronger temperature field *θ*(*ζ*) and more thermal layer thickness. Fig 15 depicts that larger Biot number *γ* produces stronger convection which leads to higher temperature field *θ*(*ζ*) and more thermal layer thickness. Table 4 is calculated in order to analyze the skin-friction coefficients $-C_{fx}\text{R}{\text{e}}_{x}^{1/2}$ and $-C_{fy}\text{R}{\text{e}}_{x}^{1/2}$ for varying *λ*, *F*_*r*_, Ω and *ϕ*. It has been observed that skin-friction coefficients $-C_{fx}\text{R}{\text{e}}_{x}^{1/2}$ and $-C_{fy}\text{R}{\text{e}}_{x}^{1/2}$ have higher values for larger Ω and *ϕ* for both SWCNTs and MWCNTs. Table 5 presents heat transfer rate (local Nusselt number) $Nu_{x}\text{R}{\text{e}}_{x}^{-1/2}$ for varying *λ*, *F*_*r*,_ Ω, *ϕ* and *γ* when Pr = 6.2. It has been noticed that larger Ω, *ϕ* and *γ* corresponds to higher local Nusselt number for both SWCNTs and MWCNTs.

**Table 4:** Numerical data of skin-friction coefficients $-C_{fx}\text{R}{\text{e}}_{x}^{1/2}$ and $-C_{fy}\text{R}{\text{e}}_{x}^{1/2}$ for various values of *λ*, *F*_*r*_, Ω and *ϕ*.

				$-C_{fx}\text{R}{\text{e}}_{x}^{1/2}$		$-C_{fy}\text{R}{\text{e}}_{x}^{1/2}$	
*λ*	*F*_*r*_	*Ω*	*ϕ*	SWCNTs	MWCNTs	SWCNTs	MWCNTs
0.0	0.1	0.1	0.1	1.27840	1.22193	0.15208	0.14538
0.1				1.34138	1.28770	0.14014	0.13301
0.3				1.46042	1.41138	0.12295	0.11566
0.2	0.0	0.1	0.1	1.36712	1.31769	0.13141	0.12409
	0.2			1.43616	1.38321	0.12999	0.12274
	0.3			1.46960	1.41495	0.12931	0.12210
0.2	0.1	0.0	0.1	1.39500	1.34433	0.00000	0.00000
		0.2		1.42152	1.36884	0.25583	0.24184
		0.3		1.45004	1.39539	0.37221	0.35234
0.2	0.1	0.1	0.01	1.15450	1.14969	0.10998	0.10930
1	1	1	0.05	1.25761	1.23288	0.11880	0.11531
1	1	1	0.15	1.56752	1.48768	0.14368	0.13221

**Table 5:** Numerical data of local Nusselt number $Nu_{x}\text{R}{\text{e}}_{x}^{-1/2}$ for various values of *λ*, *F*_*r*_, Ω, *ϕ* and *γ* when Pr = 6.2.

					$Nu_{x}\text{R}{\text{e}}_{x}^{-1/2}$	
*λ*	*F*_*r*_	*Ω*	*ϕ*	*γ*	SWCNTs	MWCNTs
0.0	0.1	0.1	0.1	0.3	0.30373	0.30052
0.1					0.30123	0.29796
0.3					0.29764	0.29436
0.2	0.0	0.1	0.1	0.3	0.29940	0.29610
	0.2				0.29911	0.29582
	0.3				0.29897	0.29569
0.2	0.1	0.0	0.1	0.3	0.27195	0.27033
		0.2			0.32544	0.32059
		0.3			0.34973	0.34351
0.2	0.1	0.1	0.01	0.3	0.26454	0.26365
			0.05		0.28625	0.28369
			0.15		0.30594	0.30225
0.2	0.1	0.1	0.1	0.0	0.00000	0.00000
				0.1	0.10638	0.10561
				0.2	0.20592	0.20404

## 6. Conclusions

Darcy-Forchheimer three-dimensional flow of carbon nanotubes subject to rotating frame and convective condition has been discussed. Main findings of presented study are listed below

- Larger values of porosity parameter *λ* and inertia coefficient *F*_*r*_ correspond to lower velocities *f*′(*ζ*) and *g*(*ζ*).
- An increase in rotation parameter Ω shows a reduction in the velocities *f*′(*ζ*) and *g*(*ζ*) while opposite trend is noticed for temperature *θ*(*ζ*).
- Velocities *f*′(*ζ*) and *g*(*ζ*) and temperature *θ*(*ζ*) are enhanced for higher nanoparticle volume fraction *ϕ*.
- Higher Biot number *γ* lead to stronger temperature *θ*(*ζ*) and more thermal layer thickness.
- Skin-friction coefficients are enhanced for higher rotation parameter Ω and nanoparticle volume fraction *ϕ*.
- Heat transfer rate (local Nusselt number) is reduced for larger porosity parameter *λ* and inertia coefficient *F*_*r*_.
